# The rise of *Candida auris*: from unique traits to co-infection potential

**DOI:** 10.15698/mic2022.08.782

**Published:** 2022-08-01

**Authors:** Nadine B. Egger, Katharina Kainz, Adina Schulze, Maria A. Bauer, Frank Madeo, Didac Carmona-Gutierrez

**Affiliations:** 1 Institute of Molecular Biosciences, University of Graz, NAWI Graz, Graz, Austria.; 2 Field of Excellence BioHealth, University of Graz, Graz, Austria.; 3 BioTechMed Graz, Graz 8010, Austria.

**Keywords:** Candida auris, candidemia, fungal infections, yeast, biofilm, resistance, COVID-19, SARS-CoV2, pandemic, co-infection, nosocomial infection, global warming

## Abstract

*Candida auris* is a multidrug resistant (MDR) fungal pathogen with a crude mortality rate of 30-60%. First identified in 2009, *C. auris* has been rapidly emerging to become a global risk in clinical settings and was declared an urgent health threat by the Centers for Disease Control and Prevention (CDC). A concerted global action is thus needed to successfully tackle the challenges created by this emerging fungal pathogen. In this brief article, we underline the importance of unique virulence traits,including its easy transformation, its persistence outside the host and its resilience against multiple cellular stresses, as well as of environmental factors that have mainly contributed to the rise of this superbug.

The clinical use of antibiotics undoubtedly ignited a new era in modern medicine: many infectious diseases, until then, the leading cause for morbidity and mortality, became systematically treatable. At the same time, this medical breakthrough drastically changed the social and political awareness of the hazards posed by various pathogens, leading to the illusion that we are generally protected from the devastating nature of infectious diseases. However, within a few decades, antibiotic resistance has increased to a level that has left some infections *de facto* untreatable. In addition, nature may not provide a sufficient number of so far unexplored diverse antibiotics, as long-standing notions have suggested. Finally, novel so far less prominent infectous agents have been emergingat an unprecedented speed. In fact, the WHO already warned in its 2007 World Health Report that infectious diseases were spreading and emerging faster than at any time in history [1]. Altogether, these challenges are a serious reminder of our persisting vulnerability against infectious diseases, and certainly, we will have to face and handle comparable menaces in future. One such challenge will be the rise of fungal infections (FIs), which have long been overviewed and neglected by healthcare authorities.

Like no other fungal pathogen, *Candida auris* has quickly unfolded to become an acutely worrisome infective agent, reaching pandemic proportions and cementing its condition as a superbug just within a decade after its first isolation in 2009 [2, 3]. Although later retrospective studies of culture collections have revealed a much earlier occurrence of *C. auris* infections dated back to the mid 1990's [2, 3], it has just been in the past few years that this pathogen has turned from a sporadic infective agent to an emerging cause of nosocomial outbreaks all over the world. An astonishing aspect about the rapid emergence of *C. auris* is the nearly simultaneous but independent appearance of genetically distinct clades on different continents [2, 3]. Four major *C. auris* clades have been identified that originally clustered by geography but are now found all over the globe: the South Asian Clade (clade I), the East Asian Clade (clade II), the South African Clade (clade III), and the South American Clade (clade IV) [2, 3]. Only recently, in 2018, a potential fifth clade, which is exclusively found in Iran (Iranian Clade), was determined [4, 5].

*C. auris* is the first and so far only fungal pathogen categorized as an urgent health threat by the Centers for Disease Control and Prevention (CDC), a category formally reserved for drug-resistant bacteria [6]. Early after the first identification of *C. auris* and due to its rapid international emergence, the CDC issued a clinical alert in 2016 to healthcare facilities, making it mandatory to report infections with *C. auris* [2, 7] but stopped global tracking of cases in February 2021 given how widespread *C. auris* had become [8]. In some settings, the epidemiology of *Candida* infections has changed drastically: while *Candida albicans* continues to be the leading cause for candidiasis, the proportion of infections by non-albicans *Candida* (NAC) *spp*. has drastically shifted towards *C. auris*, which now surpasses other prominent NAC *spp*. such as *Candida glabrata* or *Candida tropicalis* [3]. The potential of *C. auris* to become even more prominent in overall *Candida* infecitons is exemplified in a recent study, in which *C. auris* was reported to be the most predominant causative agent of *Candida* bloodstream infections (candidemia) - approximately 43% of all *Candida* isolates - in a tertiary care multispecialty center in Western India [9]. Alarmingly, all identified *C. auris* isolates within this study were resistant to fluconazole and about one third also against amphotericin B (AMB) [9], two of the most important antifungal treatment options available. A crucial characteristic of many *C. auris* strains is antifungal resistance: 90% of the isolates are reported to be resistant to at least one class of antifungal agents, 30% to at least two different classes, and even pan-resistant isolates (isolates resistant to all antifungal classes currently used in the clinics) have been described [10, 11]. This feature is especially distressing, given the limited availability of antifungal strategies. Current treatment options are rather limited due to the eukaryotic nature of fungi and only encompasses three chemical distinct classes [12]. Resistance patterns of *C. auris* accompanied by the preventive subscription and excessive use of antifungals in agriculture have most likely contributed to the sudden spread of this pathogen. Thus, the appropriate treatment of *C. auris* infections remains challenging, and novel, innovative therapeutic options are urgently needed.

An important feature of *C. auris* that explains its success as a global spreader is its easy transmission. Unlike other members of the *Candida* family, which are often associated with the human host and for which most infections have thus an endogenous origin, *C. auris* is frequently transmitted from person to person through direct or indirect contact. *C. auris* seems to be well adapted to survive outside the human host and is thus commonly isolated from human skin or the (hospital) environment [3, 13]. Notably, *C. auris* is able to persist on biotic or abiotic surfaces for several weeks (or even months) and is even capable of sustaining long periods of desiccation [2]. Moreover, thermal tolerance (*C. auris* can grow well at 42°C) and tolerance to several common disinfectants add a further layer of difficulty, especially in hospital settings [2, 3, 7]. Indeed, recent outbreaks in healthcare facilities have been linked to individuals that were colonized with *C. auris* but remained asymptotic or contaminated reusable equipment such as axillary temperature probes [4, 14].

Biofilm formation is an important virulence trait of fungal pathogens, conferring protection against various extrinsic insults and serving as a reservoir for the pathogen. *C. auris* has been reported to form high-burden, dense multilayer biofilms on skin surface, which may explain its ability to successfully colonize and persist on human skin and spread easily among patients [7, 15]. A recent study found that *C. auris* gained the ability for filamentation after passage through a mammalian body and described three distinct pheno-types: yeast, filamentation-competent yeast and filamen-tous-form cells, the latter being morphologically similar to true hyphae from *C. albicans* [16]. Intriguingly, filamentation in filamentous-form cells was supressed at 37°C, the human physiological temperature, but was promoted at lower temperature (25°C) [16]. Notably, this might explain the ability of *C. auris* to form robust biofilms on human skin, a niche generally cooler than the body core temperature, and to persist in the (hospital) environment. At the same time, when using standard RPMI medium, biofilms produced by *C. auris* are generally thinner, less complex and not as robust as biofilms formed by *C. albicans*. This observation has been attributed to a lack of true hyphae in *C. auris* biofilms under these standardized conditions; thus, for a long time, it has been hypothesized that *C. auris* is not capable of forming true hyphae at all [2, 3, 15, 17] However, growing evidence suggests that hyphal growth of *C. auris* is possible under specific circumstances, triggered by cues that are mostly different from those known for *C. albicans* filamentation (e.g. high salt concentration) [2]. This dissimilarity in hyphal growth triggers might reflect the commensal versus non-commensal nature of *C. albicans* and *C. auris*, respectively. In addition, a unique feature of *C. auris* is the aggregative phenotype, which describes the ability to form large aggregates or so called pseudohyphal-like cells, where mother and daughter cells remain attached to each other. Compared to their non-aggregated counterparts, these cells seem to be less virulent in infection models but can evade the immune system and show reduced susceptibility to antifungal treatment [2].

In comparison to other members of the *Candida* family, *C. auris* requires a compromised host immune system and thus, comorbidities such as an immunosuppressed state, advanced age, recent surgery, diabetes, application of indwelling medical devices, or the use of broad-spectrum antibiotics or antifungals (just to name a few) are major drivers of infection [2, 3]. In line, various reports indicate that also COVID-19 may be a risk factor for fungal infections [18–20]. Indeed, candidemia incidences are comparably higher in COVID-19 patients than in non-COVID-19 patients [8][21][22]. The weakened immune system due to the underlying viral infection as well as applied antiviral therapies may thereby result in higher vulnerability of COVID-19 patients [22]. Especially in healthcare settings, this seems to provide a perfect breeding ground for prolonged *C. auris* outbreaks, and several case studies all over the globe document co-infection of COVID-19 and *C. auris* in intensive care units (ICUs), resulting in devastating outcomes with mortality rates of up to 80%, despite adequate antifungal therapy [4, 18–20]. Furthermore, breakdown of routine infection prevention protocols, limited availability as well as prolonged use and/or reuse of protective equipment such as gloves or gowns, and/or alterations in cleaning and disinfection practices during the pandemic may have contributed to the spread of hospital-acquired pathogens such as *C. auris* [4, 14, 22].

Besides the unique characteristics of *C. auris*, a number of environmental factors may have contributed to the rise of this pathogen. For instance, global warming has been suggested to be key for the rapid emergence of *C. auris* from a putative plant saprophyte in specialized ecosystems that was adjusted to thermal conditions below the hu-man physiological temperature to a life-threatening human pathogen that has adapted to grow at the body temperature and has developed a significant thermotolerance [3, 23, 24]. Our modern lifestyle is constantly changing the environment, creating new habitats for infective agents and facilitating the spread of new pathogenic microbes [25]. Climate change supports the adaptation of microbes to higher temperatures, and the human basal temperature, which, so far, served as a thermal restriction zone for most fungi, will no longer provide this natural antifungal barrier with the same efficacy [23]. In that sense, *C. auris* is only a harbinger of the challenges we will have to face in the near and far future - and a call for immediate political and economic attention to much needed investment into basic and applied research of fungal biology and antifungal therapies.
